# Analyzing Social Media to Explore the Attitudes and Behaviors Following the Announcement of Successful COVID-19 Vaccine Trials: Infodemiology Study

**DOI:** 10.2196/28800

**Published:** 2021-08-12

**Authors:** Jean-Christophe Boucher, Kirsten Cornelson, Jamie L Benham, Madison M Fullerton, Theresa Tang, Cora Constantinescu, Mehdi Mourali, Robert J Oxoby, Deborah A Marshall, Hadi Hemmati, Abbas Badami, Jia Hu, Raynell Lang

**Affiliations:** 1 School of Public Policy and Department of Political Science University of Calgary Calgary, AB Canada; 2 Department of Economics University of Notre Dame Notre Dame, IN United States; 3 Department of Medicine Cumming School of Medicine University of Calgary Calgary, AB Canada; 4 Department of Community Health Sciences Cumming School of Medicine University of Calgary Calgary, AB Canada; 5 Department of Pediatrics University of Calgary Calgary, AB Canada; 6 Haskayne School of Business University of Calgary Calgary, AB Canada; 7 Department of Economics Faculty of Arts University of Calgary Calgary, AB Canada; 8 Department of Electrical and Computer Engineering Schulich Faculty of Engineering University of Calgary Calgary, AB Canada

**Keywords:** coronavirus, COVID-19, public health, social media, Twitter, behavior, risk reduction, attitudes, social network analysis, machine learning

## Abstract

**Background:**

The rollout of COVID-19 vaccines has brought vaccine hesitancy to the forefront in managing this pandemic. COVID-19 vaccine hesitancy is fundamentally different from that of other vaccines due to the new technologies being used, rapid development, and widespread global distribution. Attitudes on vaccines are largely driven by online information, particularly information on social media. The first step toward influencing attitudes about immunization is understanding the current patterns of communication that characterize the immunization debate on social media platforms.

**Objective:**

We aimed to evaluate societal attitudes, communication trends, and barriers to COVID-19 vaccine uptake through social media content analysis to inform communication strategies promoting vaccine acceptance.

**Methods:**

Social network analysis (SNA) and unsupervised machine learning were used to characterize COVID-19 vaccine content on Twitter globally. Tweets published in English and French were collected through the Twitter application programming interface between November 19 and 26, 2020, just following the announcement of initial COVID-19 vaccine trials. SNA was used to identify social media clusters expressing mistrustful opinions on COVID-19 vaccination. Based on the SNA results, an unsupervised machine learning approach to natural language processing using a sentence-level algorithm transfer function to detect semantic textual similarity was performed in order to identify the main themes of vaccine hesitancy.

**Results:**

The tweets (n=636,516) identified that the main themes driving the vaccine hesitancy conversation were concerns of safety, efficacy, and freedom, and mistrust in institutions (either the government or multinational corporations). A main theme was the safety and efficacy of mRNA technology and side effects. The conversation around efficacy was that vaccines were unlikely to completely rid the population of COVID-19, polymerase chain reaction testing is flawed, and there is no indication of long-term T-cell immunity for COVID-19. Nearly one-third (45,628/146,191, 31.2%) of the conversations on COVID-19 vaccine hesitancy clusters expressed concerns for freedom or mistrust of institutions (either the government or multinational corporations) and nearly a quarter (34,756/146,191, 23.8%) expressed criticism toward the government’s handling of the pandemic.

**Conclusions:**

Social media content analysis combined with social network analysis provides insights into the themes of the vaccination conversation on Twitter. The themes of safety, efficacy, and trust in institutions will need to be considered, as targeted outreach programs and intervention strategies are deployed on Twitter to improve the uptake of COVID-19 vaccination.

## Introduction

The COVID-19 pandemic is emerging as one of the greatest public health threats in history, with over 140 million infections and 3 million deaths worldwide attributed to the SARS-CoV-2 virus as of April 2021 [1]. As transmission of COVID-19 continues around the globe, a COVID-19 vaccine is an important and valuable tool to reduce the spread of infection. Due to the critical need, the speed of vaccine development, production, and mass rollout has been faster than ever seen before, leading to concerns about vaccine efficacy and safety [2,3]. The intricacy surrounding COVID-19 vaccine hesitancy appears to be further reaching and fundamentally different than other immunizations [2]. Vaccine production cannot meet demand, requiring rollout plans for targeted subpopulations, and there are several types of COVID-19 vaccines being used within one country [2]. This has led to increased concern, mistrust, and confusion surrounding COVID-19 vaccination.

Despite the massive undertaking of vaccine development, a vaccination program is only as successful as its uptake. Vaccine hesitancy, defined as a delay in acceptance or refusal of vaccines despite their availability [4], was mentioned by the World Health Organization as one of the top 10 threats to public health in 2019 [5]. The effective rollout of a COVID-19 vaccine strategy may be obstructed by the beliefs and attitudes of vaccine-hesitant individuals worldwide [6]. A recent survey of US adults in April 2020 found that 23% of persons would not be willing to get vaccinated against COVID-19 [7]. In Canada, a survey in March 2021 demonstrated that 76.9% of Canadians were very or somewhat willing to receive a COVID-19 vaccine [8].

Vaccine hesitancy is a “multifaceted, deeply complex construct that may be rooted in the moral composition that guides our daily decision making” [8,9]. Studies have identified that vaccination decisions are shaped by multiple complex interactions between individual, community, cultural, historical, political, and societal factors [2,10]. There have been several metrics and scales developed and used to measure vaccine confidence and hesitancy [11-13]. The Vaccine Confidence Index (VCI) survey tool was developed to measure individuals’ perceptions on the safety, importance, effectiveness, and religious beliefs toward vaccines, which were identified as key drivers of public confidence in vaccination [11]. Another validated score measuring the psychological antecedents of vaccination is known as the 5C scale [13]. The 5C scale includes confidence (trust and attitudes), complacency (not perceiving diseases as high risk), constraints (structural and psychological barriers), collective responsibility (willingness to protect others), and calculation (information searching) [13].

Negative beliefs about vaccines may prevent the implementation of provaccination policies. Public health officials need to prioritize implementing strategies to help reduce these negative perceptions [14]. Traditional approaches to promoting immunization have assumed that inadequate knowledge of the associated risks and benefits drive hesitancy; however, this stance has proven to be ineffective as an intervention [15]. The source of the hesitancy is often both from lack of information and from lack of trust in institutions such as the government, physicians, and pharmaceutical companies [16,17]. Rather than just trying to enhance knowledge, a different approach to overcoming vaccine hesitancy for COVID-19 may be to focus on changing personal attitudes [15].

Prior studies have demonstrated that social media can help understand attitudes and behaviors during public health crises and promote health messaging [18-20]. Particularly, Twitter has been used for public health research and more specifically regarding COVID-19 [18,21]. A recent study aimed to characterize the main topics of Twitter conversations related to COVID-19 and identified four main themes including the origin of the virus, its sources, the impact on people/countries/economy, and lastly methods of mitigating the risk of infection [18].

Attitudes toward vaccination are, in large part, shaped by information and ideas individuals encounter through social media [22,23]. Social media is a principal informational forum for vaccination uptake with large proportions of content involving antivaccination messaging [22,24]. Reliable and accurate information on social media is often mixed with inaccurate, conspiratorial, incomplete, or biased messages [22]. A recent study evaluating vaccine hesitancy through content analysis of tweets in Canada identified major themes including safety, suspicion of economic or political motivation, knowledge deficit, opinions of authority figures, and lack of liability from pharmaceutical companies [25]. This study demonstrates the significant utility of using Twitter to better understand vaccine hesitancy at a population level [25]. However, a broader reaching and deeper understanding of current patterns of communication that characterize the immunization debate on social media platforms is needed to inform public health interventions aimed toward influencing attitudes on immunization [15,17,22,23].

This study used social network analysis (SNA) and unsupervised machine learning to characterize COVID-19 vaccine content on Twitter, which allows researchers to access the application programming interface (API). The use of machine learning in social sciences is expanding and has generated interesting methodological conversations on different approaches to study COVID-19 sentiment, attitude, and emotion [26-28]. With this study, we aimed to use social media content analysis to provide a further understanding of societal attitudes, communication trends, and barriers to COVID-19 vaccine uptake, at a critical time during the COVID-19 pandemic, just following reporting of initial vaccination trials. We hypothesize that these data will be critical globally for developing targeted outreach programs and intervention strategies on Twitter to impact COVID-19 vaccine hesitancy.

## Methods

### Study Data

Tweets published in English and French using specific words (“COVID” AND (“vacc” OR “vax” OR “immu”)) in either the content of the tweet or hashtag derivatives were collected through the Twitter API between November 19 and 26, 2020. This Boolean query was selected in order to maximize inclusivity without adding unnecessary noise to our data set. For example, with the query “vacc,” we effectively targeted all derivatives such as “vaccine,” “vaccines,” “vaccinated,” “vaccination,” “vaccinations,” and associated hashtags. Furthermore, it follows search queries from similar studies examining vaccine hesitancy and social media [29-31]. English and French were selected as they are two of the most common languages used on social media. The data set included several features, such as descriptive information about the user, username, content of tweets (hashtags, relationship among users such as retweet, replies, and mentions, etc), self-reported location of the user, number of followers, date of account creation, and time of tweet posting. Tweets were extracted using Twitter’s public streaming API allowing researchers to collect a random sample of tweets in real time of up to 1% of all public tweets published daily.

This time period was crucial in COVID-19 vaccination conversations on social media as it came a week after pharmaceutical companies (Pfizer and Moderna) announced successful trials of their COVID-19 vaccines [32,33]. For the first time since the beginning of the COVID-19 pandemic in early 2020, social media conversations on vaccination were based, at least in part, on plausible empirical information about the efficacy and availability of a vaccine. We therefore wanted to evaluate the public’s initial reaction and response to COVID-19 vaccination. Few prior studies have included this time frame during the analysis period [30,34-36].

### Social Network Analysis

In this study, we designed a data analytics workflow by first using SNA to identify social media accounts most likely expressing doubtful or mistrustful opinions on COVID-19 vaccination. This network was created using a weighted retweet directed network to represent connections between accounts. Although retweets are not a perfect indicator of like-mindedness, on aggregate, users have a proclivity to engage more with accounts that reflect some form of social or intellectual homophily [37]. Through SNA, we examined the underlying structure of community clustering within the broader social network exposed by online interactions, isolating different “communities” of like-minded users. The Louvain modularity method [38] was used to detect subclusters of online communities mentioning COVID-19 vaccination.

### Natural Language Programming Analysis

Based on our SNA results, we developed an unsupervised machine learning approach to natural language processing by using a sentence-level algorithm transfer function to detect semantic textual similarity [15]. Our goal was to examine how antivaccine conversation clusters talk about or frame a possible COVID-19 vaccine without prior assumptions about the nature of the conversation. For this analysis, we first tokenized our sentences (tweets) and cleaned the data set, removing duplicates, stop words, symbols, numbers, punctuation, URLs, whitespaces, and stemmed words to their roots. We also added a language identifier to remove tweets in languages other than French or English and reduce noise in the data set. We then used DistilBERT [39], a knowledge distillation learning model, for sentence-level embedding. DistilBERT is a compressed version of BERT (Bidirectional Encoder Representations from Transformers), which retains much of the computational accuracy of BERT without the environmental costs associated with high-dimensionality embeddings. DistilBERT positions all sentences (here tweets from the antivaccine conversation) in a multidimensionality vector space from which we can compare the semantic similarity of tweets.

**Figure 1:**
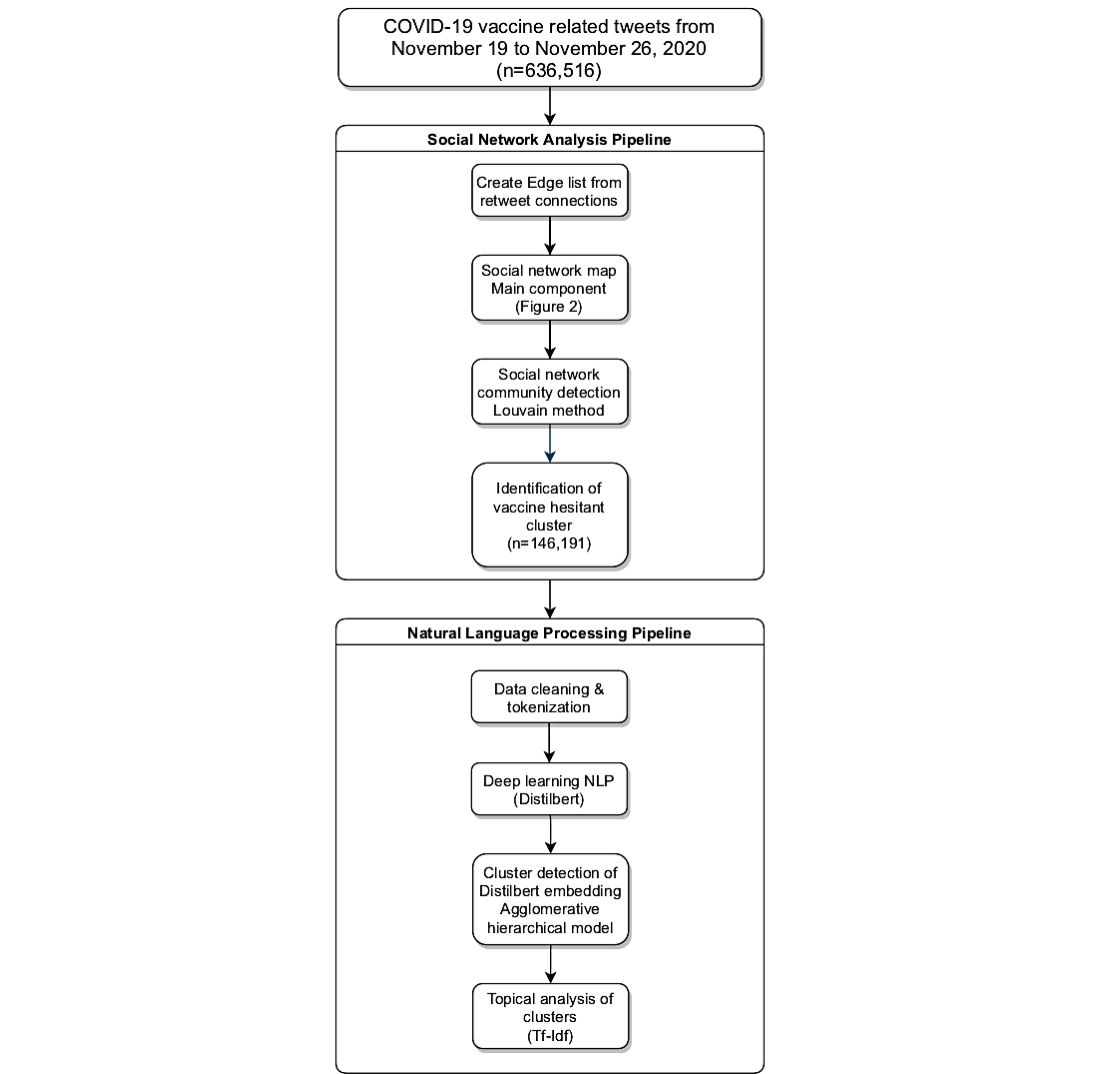
Data analytics workflow. NLP: natural language processing.

We used an agglomerative hierarchical cluster model to identify a relevant number of clusters from the multidimensionality output produced by the DistilBERT computation, which provided us a measure to identify different topics of similar tweets. To infer topics from our clustering modeling, we used a bi-gram of term frequency-inverse document frequency (tf-idf), which measures the originality of a word by comparing the number of occurrences of the word in a document (term frequency) and the number of documents with the word (inverse document frequency). This measure allows us to undervalue words that appear frequently in most documents (such as “the”) and provide little information, and overvalue words that appear sporadically in the corpus, but often in some documents. Topics were then inferred manually based on the cluster model and informed by the tf-idf output. Figure 1 illustrates our data analytics pipeline.

## Results

**Figure 2:**
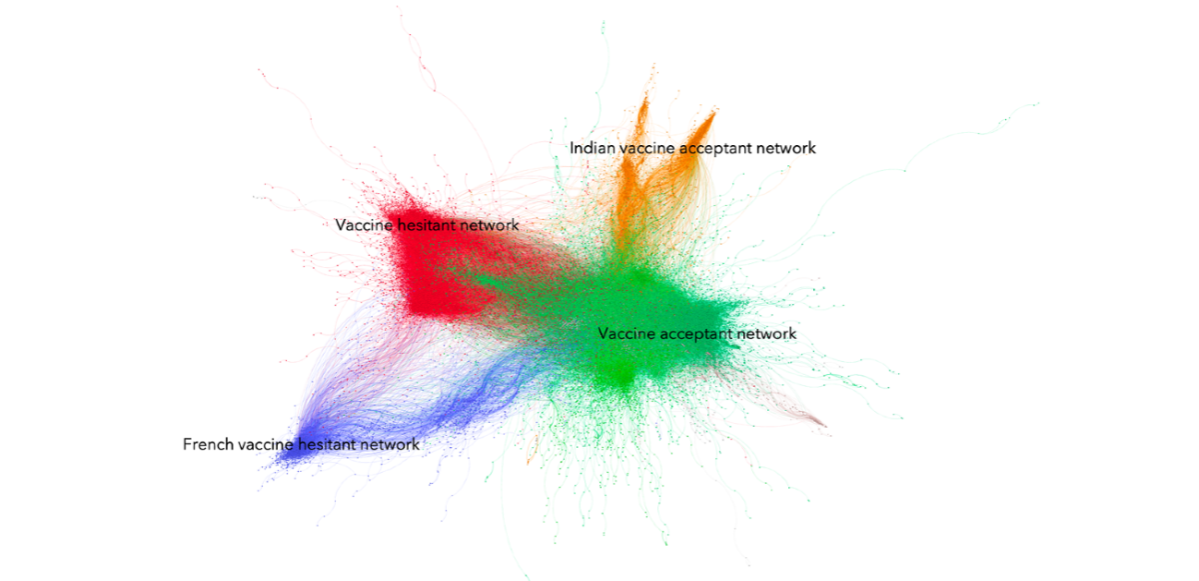
Twitter retweet cluster of COVID-19 vaccination (November 19 to November 26, 2020). The vaccine acceptant cluster (n=211,549), vaccine hesitant cluster (n=88,892), Indian vaccine acceptant cluster (n=28,713), and French vaccine hesitant cluster (n=11,509) are seen. Nodes represent specific Twitter accounts, while edges represent retweet activity between accounts. Presented are the four largest online communities mentioning COVID-19 vaccination.

In total, 636,516 tweets were collected from 428,535 accounts, for an average of 79,564 tweets per day. Figure 2 presents the cluster map of COVID-19 immunization conversations on Twitter between November 19 and November 26, 2020. We found a polarized conversation about immunization on social media.

During this observation period, a large proportion of accounts debating COVID-19 immunization on Twitter were connected and exposed to social media conversations promoting vaccination narratives. The largest cluster (green), comprising approximately 49.4% (n=211,549) of Twitter accounts, revealed a vaccine acceptant point of view. Based on degree centrality, the cluster seemed to overlap with more progressive-leaning political leaders, such as Hillary Clinton and US Representative Alexandria Ocasio-Cortez (D-14-NY), and mainstream news media, such as the NY Post, the Hill, ABC, and Reuters. A second provaccine cluster (orange) centered on the Indian COVID-19 immunization online debate, with political leaders, such as Rahul Gandhi and Press Trust of India (the largest news agency in India), leading the conversation.

As Figure 2 indicates, we also found two clusters opposite to these vaccine acceptant clusters exhibiting more vaccine hesitant narratives. There were 23.4% (n=146,191) of conversations on Twitter during this period of observation that can be directly attributed to vaccine hesitancy. First, in red, our study identified a large cluster comprising 88,892 Twitter handles accounting for 18.4% of all accounts in the cluster. These interactions from the Twittersphere in English originated mostly from the United States, the United Kingdom, and Canada, and gravitated around accounts of prominent antivaccine physicians and organizations, right-wing activists, show hosts, such as Rush Limbaugh in the United States and Simon Dolan or Michael J Blair in the United Kingdom, and some alternative news organizations, such as Breitbart News. In this conversation cluster, we found an overlap between ideologically leaning advocates, especially those associated at the margins of right-wing or Conservative parties, and antivaccine online conversations. We found considerable cross-pollination between accounts originating from all across the English Twittersphere, demonstrating the internationalization of COVID-19 vaccination conversations on social media. Second, in blue, a smaller cluster representing 2.7% (n=11,509) of accounts appeared to be shaped around Francophone (from France and Québec) vaccine hesitant conversations.

Examining the COVID-19 vaccine hesitancy narrative during this observation period provided key insights on what drives attitudes. As shown in Table 1, our unsupervised machine learning analysis identified 12 specific archetypical vaccine hesitancy tweets (we tested a different number of clusters between 9 to 20). Social media content highlighted concerns over the efficacy and the safety of a possible COVID-19 vaccine fitting into the 5C scale domain of confidence. Five broad categories focused on vaccine efficacy in our data set. The first topic (topic 3 in Table 1) regrouped tweets suggesting that attempts to produce a COVID-19 vaccine, especially using mRNA technology, remain tentative. These tweets highlighted our failure to develop an HIV vaccine with mRNA and that existing mRNA vaccines for COVID-19 have not been tested enough to demonstrate efficacy. The second category of vaccine hesitancy tweets concentrating on efficacy (topic 2) focused on a comment made by Sir John Irving Bell on November 20, 2020, that existing vaccines were unlikely to completely rid the population of COVID-19. The third and fourth vaccine efficacy topics (topics 7 and 8) grouped together tweets from prominent physicians arguing that existing polymerase chain reaction testing is flawed and that there are no indications of long-term T-cell immunity for COVID-19. The last topic highlighted (topic 10), which obtained subsequent media attention, suggested that as much as 25% of the population would have contracted COVID-19 by the time a vaccine would be rolled out, and consequently, the vaccine would be unnecessary to reach herd immunity.

As for the concern of vaccine safety, we found two broad groups of tweets. The first (topic 5) highlighted a classic antivaccine story where an emergency medical technician/fire rescue was required to get TDAP (tetanus, diphtheria, and pertussis) boosters and had complications. Although not specifically addressing the issue of a COVID-19 vaccine, these tweets emphasize how existing antivaccine narratives have created a baseline from which some individuals frame a COVID-19 vaccine. The second topic (topic 9) centered on tweets in French framing a COVID-19 vaccine as a poison and suggesting that mRNA technology has not been tested yet and would be harmful.

Although safety and efficacy concerns remain a major component explaining COVID-19 vaccine hesitancy, it is only half of the story. As we examined the content of tweets of users integrated in the vaccine hesitancy online cluster, we found a large proportion of those interactions that emphasized a concern for personal freedom and/or some form of mistrust of institutions. In the 5C scale of psychological antecedents of vaccination, confidence in vaccines includes trust in the system that delivers the vaccines and the motivation of policy makers who decide on the need of vaccines [13]. Tweets were framed in three directions. First, a large percentage of such tweets (n=45,628, 31.2%) expressed some criticism toward the government’s handling of the COVID-19 crisis, especially decisions to curb individual freedom. COVID-19 immunization is framed as the right of individuals to decide for themselves whether or not to be vaccinated. Any indication that governmental authorities might require vaccination is perceived as a direct attempt to limit individual freedom. Two main topics (topics 1 and 12) highlighted mistrust in government policy, with one condemning the Johnson government in the United Kingdom for proposing that individuals vaccinated for COVID-19 could receive “freedom passes” and one criticizing Denmark’s decision to cull its mink population to halt the spread of a coronavirus variant. Some topics (topics 6 and 11) expressed mistrust in multinational corporations, most notably airline companies, such a Qantas, who suggested that vaccination should be made compulsory for international travel. This decision is framed as a direct restriction of personal freedom by multinational corporations, and some users made a direct reference to populist narratives suggesting that these policies would only affect commercial flights and elites would be able to avoid vaccination while using private flights.

**Table 1:** Inferred topic analysis of the COVID-19 vaccination hesitancy cluster (November 19 to November 26, 2020).

Topic	Topic keywords bi-gram (tf-idf)	Inferred topic	Tweets (N=146,191), n (%)
1	Countries test, results multinational, multinational companies, the Johnson, commie, passes two, proposing freedom, commie proposing, full commie, Johnson government, gone full, government gone	Trust in the government	33,578 (23.0%)
2	Tanked economy, tweet day, bell, disturbing tweet, day professor, most disturbing, bell talking, professor sir, irving bell, john irving, irving, sir john	COVID-19 vaccine hesitancy: Efficacy	30,576 (20.9%)
3	Produced cell, unlicensed produced, lines aborted, shots wont, HIV/AIDS, won’t walk, test enough, infection enough, antibodies past, enough effective, enough antibodies, past infection	COVID-19 vaccine hesitancy: Efficacy	28,818 (19.7%)
4	Presi, COVID literally, one two, presi im, corner presi, literaly around, im sitting, sitting thinking, thinking incredible, incredible one	Support for Trump’s management of the COVID-19 crisis	11,631 (8.0%)
5	nick healthy, jer, emtfire, gau nick, jer gau, damage jer, dayprofessor sir, tweet dayprofessor, dayprofessor	COVID-19 vaccine hesitancy: Side effects	9579 (6.6%)
6	Passports we, elite continue, the elite, commercial flights, fly private, breaking qantas, ceo confirms, you’ve vaccinated, compulsory international, international, confirms proof, proof you’ve	Trust in multinational corporations	9286 (6.4%)
7	Attacked, attacked since, since apr, apr highlighting, highlighting imp, viciously, imp tcell, sarscov, despite published, published COVID, viciously attacked, ive viciously	COVID-19 vaccine hesitancy: Efficacy	7896 (5.4%)
8	Label new, staff form, group label, jabs poison, longterm tcell, immunity cases, watch interview, interview dr, science longterm, discussing hysteria, phd discussing, dr phd.	COVID-19 vaccine hesitancy: Efficacy	6921 (4.7%)
9	Arn caca, quand, faire, arn, bonjour, confinement vaccin, avec, caca, confinement, vaccin contre, contre le, le vaccin	COVID-19 vaccine hesitancy: Side effects	2822 (1.9%)
10	Coronavirus im, pr quarter, newspapers, im delighted, delighted mainstream, mainstream newspapers, newspapers picking, picking pr.	COVID-19 vaccine hesitancy: Efficacy	2320 (1.6%)
11	Fly must, want fly, begins if, must take	Trust in multinational corporations	1586 (1.1%)
12	Protestors, force in, could force, COVID law, authorities could, Denmark, proposed forced, protesting proposed, protestors protesting, in Denmark, Denmark protestors, law authorities	Trust in the government	1178 (0.8%)

A final residual category (topic 4) in the vaccine hesitancy conversation singled out a tweet by radio host Rush Limbaugh mentioning that two COVID-19 vaccines were now approved and praising President Trump’s management of the crisis. This tweet was widely circulated in the vaccine hesitant conversation cluster and underlines the reality that any conversation on COVID-19 immunization, and to some degree, vaccine hesitancy clusters on social media, intersect with broader clusters structured by current political polarization.

**Figure 3:**
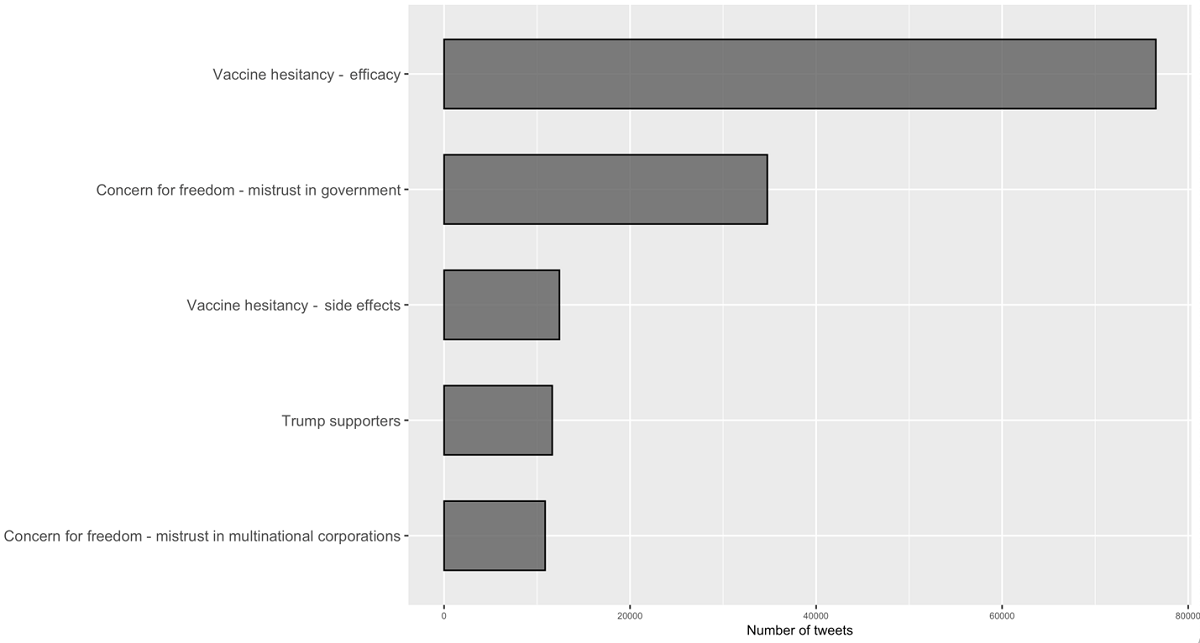
Distribution of inferred topics in COVID-19 vaccine hesitancy clusters on social media. This figure presents the aggregated results of inferred topics in vaccine hesitancy clusters from Table 1.

Figure 3 presents the approximate distribution of these inferred topics in COVID-19 vaccine hesitancy clusters identified during this observation period and allows us to understand what shapes an individual’s attitude toward a COVID-19 vaccine. First, as this data set was collected right after the news of positive trials of COVID-19 vaccines by Pfizer and Moderna, more than half of the tweets (n=76,531, 52.4%) mentioned vaccine efficacy and raised suspicions on whether creating vaccines using mRNA technology was achievable or whether immunization would be long-lasting. With respect to vaccine safety, only 8.5% (n=12,401) of the social media debate in vaccine hesitancy clusters doubted its safety. Second, nearly one-third (n=45,628, 31.2%) of conversations on vaccine hesitancy clusters on Twitter expressed concerns for freedom or mistrust of institutions (either the government or multinational corporations). These results suggest that one key determinant of vaccine hesitancy is trust in institutions. It suggests that vaccine confidence building is a problem of shaping attitudes toward COVID-19 vaccines, which falls into the realm of health policy, as well as promoting social/political trust, which falls more into the realm of politics.

## Discussion

### Principal Findings

We analyzed more than 600,000 tweets over 1 week just following the announcement of successful COVID-19 vaccine trials, to characterize and understand global public perceptions and attitudes surrounding the COVID-19 vaccine and themes driving vaccine hesitancy. Our analysis revealed contrasting conversations about COVID-19 immunization on social media with both vaccine acceptant and vaccine hesitant clusters. Identified were the main themes driving the vaccine hesitant conversation at that time, including concerns of safety, efficacy, and freedom, and mistrust in institutions (either the government or multinational corporations). A main theme was the safety and efficacy of mRNA technology and side effects. The conversation around efficacy was that vaccines were unlikely to completely rid the population of COVID-19, polymerase chain reaction testing is flawed, and there is no indication of long-term T-cell immunity for COVID-19. Nearly one-third (31.2%) of the conversations on COVID-19 vaccine hesitancy clusters expressed concerns for freedom or mistrust of institutions (either the government or multinational corporations) and nearly a quarter (23.8%) expressed criticism toward the government’s handling of the pandemic.

The main themes identified in this study fall under the domain of confidence using the 5C scale. Confidence is a measure of a person’s level of trust in vaccine safety and efficacy, as well as in those involved in vaccine administration including policy makers and health professionals. Studies have shown that confidence is positively correlated with attitudes toward vaccination, knowledge of vaccination, and trust in health care, while it is negatively correlated with conspiracy mentality and medical harms [13]

The speed of development, production, and mass rollout of a COVID-19 vaccine has been unprecedented and has led to concerns around the safety and efficacy of vaccination. A common theme identified at that time was concern regarding mRNA technology. mRNA-based therapeutics have been used for cancer vaccines in the past; however, compared to other vaccine technology, it has not been clinically tested to the same extent [40]. Dror et al published results of a survey in which 70% of the general public responded with concerns about the safety of the COVID-19 vaccine [41]. We identified that less than 10% of the social media debate in vaccine hesitancy conversations during our observation period doubted its safety. Nevertheless, as COVID-19 vaccines are rolled out, it is highly probable that antivaccine social media conversations will transition from arguing about efficacy to questioning vaccine safety.

Mistrust in institutions has emerged as a predominant theme of vaccine hesitancy conversations on Twitter. Prior literature has reported mistrust in doctors, government sources, and pharmaceutical companies as reasons for hesitancy [16]. Governments are directly involved in many aspects of vaccine development, from funding to eventual safety approval. Individuals who believe the government is incompetent or malicious may not trust that these functions have been carried out in an appropriate way. Trust in government covaries strongly with generalized trust in other people and feelings of connectedness to others in society [42-48]. These measures of “social capital” in turn have been linked with reduced willingness to contribute to public good [43]. For example, there is a link between government trust and willingness to pay taxes [49]. Conversely, increased ethnic or political fragmentation, which creates feelings of division in society, has been shown to reduce the quality of the government and reduce physical distancing during the COVID-19 pandemic [50,51]. Because the COVID-19 vaccine has public benefits that go beyond individual protection, individuals with low social trust may be less willing to contribute to the public good by getting the vaccine. Furthermore, recent studies using survey data have been increasingly associating trust in the government with COVID-19 behavior and vaccine hesitancy, and our social media data add to this literature [52,53].

There is extensive literature examining both individual and aggregate correlates of trust. At the individual level, trust is positively correlated with education [54] and civic engagement [42]. Aggregate measures of social trust vary with the actual performance of the government, and poor economic growth, high crime, mass protests, and political scandals appear to reduce the trust of citizens in the government [55,56]. Conversely, increasing transparency in the government appears to improve public trust in authorities [57]. Highlighted is the importance of building trust in institutions, which needs to be incorporated into models aimed at targeting vaccine hesitancy in addition to the traditional pillars of communication, information, and cognition.

Globally, persons are challenged with an overabundance of information on COVID-19 and COVID-19 vaccination, in which misinformation has been disseminated rampantly, likely fueling hesitancy [58]. Yaqub et al highlighted in a critical review of vaccine hesitancy that “hesitancy is not a rare phenomenon or confined solely to antivaccinationists; it includes people who have not yet rejected vaccination. Focusing on only vaccine uptake rates and neglecting underlying attitudes is likely to underestimate the challenge of maintaining vaccination coverage in the future” [16]. We demonstrated that social media analysis provides insights into societal attitudes, communication trends, and barriers to vaccine uptake that must be considered when developing strategies to address vaccine hesitancy.

The strength of this study lies in the methodology undertaken, which involved a bottom-up approach for the identification of cases and SNA. Previous studies have generally adopted a top-down approach to data collection, isolating known antivaccine accounts and analyzing its content and diffusion. Although critical in understanding the structure and nature of antivaccination framing on social media, such methods run the risk of selection bias. Moreover, by analyzing tweets in both French and English, we were able to broaden the scope of our vaccine conversation clusters, increasing the generalizability of this work. The noise of this analysis was minimized by linking the vaccination keywords with “COVID.”

### Limitations

There are several limitations in our work. Although social media is increasingly used as a source of information and social interaction, even on matters related to health policy, it is an environment where participants self-select themselves in the population and is not a representative sample of the general population. In this sense, our results reflect a specific conversation around COVID-19 vaccination, and studies focusing on other social media platforms (eg, Facebook, WhatsApp, Reddit, and YouTube) and more traditional news media would offer a more complete overview of how antivaccine narratives are structured. Demographic segmentation of these clusters was not possible as further background information on the individuals in each cluster was not available; however, this would be a valuable component to future research. Our analysis was done on data collected right at the onset of news confirming the successful clinical trials of COVID-19 vaccines by Pfizer and Moderna. Our research offers a baseline from which we can understand the evolution of the online debate about COVID-19 vaccination. However, our assumption is that such a conversation will evolve and change throughout the pandemic, and we should expect the saliency of antivaccine arguments (safety, efficacy, and trust in institutions) to fluctuate as new information and policies are put in place. Future research is needed to monitor the COVID-19 vaccine hesitancy conversation through adopting a dynamic approach by collecting tweets over longer time periods and analyzing the patterns of change over time. Finally, as data were collected through the Twitter streaming API, the sample we analyzed may not have been fully randomized. The Twitter streaming API tends to overrepresent central users and is influenced by Twitter’s sampling algorithm. Furthermore, given the size of the data set, we could not remove bots, which may have potentially skewed certain results.

### Conclusions

The recent global rollout of COVID-19 vaccination has brought vaccine hesitancy to the forefront in managing this pandemic. Hesitancy in accepting COVID-19 vaccination is fundamentally different from other vaccinations due to the new technologies being used, rapid development, and widespread global distribution. Attitudes on vaccines are largely driven by online information, particularly information on social media. We demonstrated that social media content and network analysis provides insights into societal attitudes, communication trends, and barriers to vaccine uptake. Identified themes driving the vaccine hesitant conversation included concerns of safety, efficacy, and freedom, and mistrust in institutions (either the government or multinational corporations). These themes will need to be considered as targeted outreach programs and intervention strategies are deployed globally in attempts to change personal attitudes on Twitter and improve the uptake of COVID-19 vaccination.

## Abbreviations

API: application programing interface
BERT: Bidirectional Encoder Representations from Transformers
SNA: social network analysis
